# A Narrative Review of Human Clinical Trials on the Impact of Phenolic-Rich Plant Extracts on Prediabetes and Its Subgroups

**DOI:** 10.3390/nu13113733

**Published:** 2021-10-22

**Authors:** Wen Xin Janice Lim, Cheryl S. Gammon, Pamela von Hurst, Lynne Chepulis, Rachel A. Page

**Affiliations:** 1 School of Health Sciences, Massey University, Auckland 0632, New Zealand; w.x.j.lim@massey.ac.nz (W.X.J.L.); C.Gammon@massey.ac.nz (C.S.G.); 2 Riddet Institute, Massey University, Palmerston North 4442, New Zealand; 3 School of Sport, Exercise and Nutrition, Massey University, Auckland 0632, New Zealand; p.r.vonhurst@massey.ac.nz; 4 Waikato Medical Research Centre, Te Huataki Waiora School of Health, University of Waikato, Hamilton 3216, New Zealand; lynne.chepulis@waikato.ac.nz; 5 School of Health Sciences, Massey University, Wellington 6021, New Zealand; 6 Centre for Metabolic Health Research, Massey University, Auckland 0632, New Zealand

**Keywords:** functional food, polyphenol, impaired glycemic control, impaired glucose tolerance, impaired fasting glucose

## Abstract

Phenolic-rich plant extracts have been demonstrated to improve glycemic control in individuals with prediabetes. However, there is increasing evidence that people with prediabetes are not a homogeneous group but exhibit different glycemic profiles leading to the existence of prediabetes subgroups. Prediabetes subgroups have been identified as: isolated impaired fasting glucose (IFG), isolated impaired glucose tolerance (IGT), and combined impaired fasting glucose and glucose intolerance (IFG/IGT). The present review investigates human clinical trials examining the hypoglycemic potential of phenolic-rich plant extracts in prediabetes and prediabetes subgroups. *Artemisia princeps* Pampanini, soy (*Glycine max* (L.) Merrill) leaf and *Citrus junos* Tanaka peel have been demonstrated to improve fasting glycemia and thus may be more useful for individuals with IFG with increasing hepatic insulin resistance. In contrast, white mulberry (*Morus alba* Linn.) leaf, persimmon (*Diospyros kaki*) leaf and *Acacia. Mearnsii* bark were shown to improve postprandial glycemia and hence may be preferably beneficial for individuals with IGT with increasing muscle insulin resistance. *Elaeis guineensis* leaf was observed to improve both fasting and postprandial glycemic measures depending on the dose. Current evidence remains scarce regarding the impact of the plant extracts on glycemic control in prediabetes subgroups and therefore warrants further study.

## 1. Introduction

Globally, diabetes rates have been increasing at an alarming rate. In 2019, it was estimated that 463 million (ages 20–79 years) (9.3%) people were living with diabetes worldwide, an increase of 62% from 2009 with the number expected to increase to 700 million (10.9%) by 2045 [1]. According to the International Diabetes Federation (IDF), the current annual global health expenditure on diabetes is estimated to be USD 760 billion and is projected to reach USD 845 billion by 2045 [1]. Much of the health costs come from the complications that are associated with diabetes, which can affect the eyes, kidneys and nervous system [2,3], and heighten the risk of cardiovascular morbidity and mortality [4].

Although the rates of Type 1 diabetes mellitus have also been the main driver of the increased rates of diabetes, it is Type 2 diabetes mellitus (T2DM) that constitutes approximately 90% of diabetes worldwide [1]. This has largely occurred in parallel with the obesity epidemic. Given the burden this is putting and will put on health systems, it is therefore crucial to identify strategies that would prevent or slow the development of T2DM.

### 1.1. Prediabetes and Its Subgroups

Prediabetes is an intermediate state of hyperglycemia with blood glucose levels above normal but not high enough to be classified as T2DM [5]. Prediabetes is a high-risk state for developing T2DM [6] and has an annual conversion rate of 5–10% [5,7]. Therefore, early detection of prediabetes in conjunction with effective interventions may reduce the risk of developing future T2DM [8,9].

There is increasing awareness that individuals with prediabetes are not a homogeneous group [10,11] and different metabolic profiles exist reflecting varying degrees of insulin resistance and β-cell dysfunction [10]. Three subgroups of glucose intolerance have been identified, which are impaired fasting glucose (IFG), isolated impaired glucose tolerance (IGT), and combined impaired fasting glucose and impaired glucose tolerance (IFG/IGT) [10,12]. These subgroups have distinctly different glycemic metabolic profiles [7,13,14,15,16,17,18,19,20,21], and exhibit different postprandial glucose (PG) and postprandial insulin (PI) shapes after a carbohydrate load [17,20,21,22,23].

According to the American Diabetes Association (ADA) guidelines, individuals with isolated IFG have elevated fasting blood glucose (FBG) of 100–125 mg/dL (5.6–6.9 mmol/L) while having a normal 2 h postprandial glucose (2hPG) of <140 mg/dL (<7.8 mmol/L) [24]. Individuals with IFG tend to exhibit increased endogenous glucose production (EGP), reduced hepatic insulin sensitivity, stationary β-cell dysfunction and/or chronic low β-cell mass, defective early phase insulin secretion while maintaining normal second phase insulin secretion with PG returning to normal after 2 h, altered glucagon-like peptide-1 (GLP-1) secretion and inappropriately elevated glucagon secretion [12,13,20,21,22,23,25,26,27,28,29,30,31,32,33]. They tend to also possess healthy or near healthy peripheral insulin sensitivity [20,25,27,29].

Individuals with isolated IGT typically have normal FBG of <100 mg/dL (<5.6 mmol/L), but an abnormally elevated 2hPG of 140–199 mg/dL (7.8–11.0 mmol/L) [24]. Characteristics specific to IGT may include increased or normal EGP, reduced peripheral insulin sensitivity, near-normal hepatic insulin sensitivity, impaired early and late phase insulin secretion with a subsequent rise in PG that is unable to return to normal baseline after 2 h, persistent and progressive loss of β-cell function, reduced secretion of gastric inhibitory polypeptide (GIP) and inappropriately elevated glucagon secretion [12,13,20,21,22,23,25,26,27,28,29,30,33,34,35].

Individuals with combined IFG/IGT fulfill both criteria of having elevated FBG of 100–125 mg/dL (5.6–6.9 mmol/L) and elevated 2hPG of 140–199 mg/dL (7.8–11.0 mmol/L) [24]. IFG/IGT takes the worse form of impaired glucose control with a reduced glucagon suppression, impaired hepatic and peripheral insulin sensitivity and progressive loss of β-cell function, with a sustained rise in PG that does not return to normal baseline after 2 h [20,21,29,36,37].

### 1.2. Proposed Mechanisms of Action of Plant Extracts on Prediabetes Subgroups

Phenolic-rich plant extracts are increasingly being recognized for their hypoglycemic effects and potential to help regulate glucose metabolism in the human body [38,39,40,41,42,43,44,45,46,47,48,49]. The complex and unique phenolic structures of the plant extracts such as the number and position of hydrogen moieties (e.g., OH) and double bonds determine their bioavailability and subsequent interaction with membrane-bound brush border enzymes, apically located transporters and receptors involved in glucose metabolic pathways [50,51,52]. The hypoglycemic properties of plant extracts provide the opportunity for their use as an alternative or to complement anti-diabetic medications with few to no adverse effects [53,54].

Anti-diabetic pharmacological drugs have targeted various organs, such as muscle, pancreas, liver and gut, responsible for glucose metabolism with specific mechanisms of action to improve glycemic control [54]. Drugs such as metformin belonging to the class of biguanide inhibit liver glucose production, whilst sulfonylureas and meglitinides that are insulin secretagogues target liver insulin resistance and therefore are utilized to treat IFG or IFG/IGT to improve fasting glycemic responses [20,55,56]. In contrast, drugs targeting peripheral or muscle insulin resistance to improve skeletal muscle insulin sensitivity such as peroxisome proliferator-activated receptor-gamma (PPAR-γ) agonists, as well as α-glucosidase inhibitors, GLP-1 agonists, dipeptidyl peptidase-4 (DPP4) inhibitors and thiazolidinediones, are most efficacious when taken together with a meal to improve postprandial glycemic responses and hence, may be better utilized by those with IGT or IFG/IGT [20].

Similarly, plant extracts have been demonstrated to modulate specific glycemic pathways in carbohydrate metabolism. Plant extracts that may protect β-cell function against glucotoxicity via activating 5′AMP-activated protein kinase (AMPK), suppress hepatic gluconeogenesis, stimulate insulin secretion and reduce hepatic insulin resistance, oxidative stress, and inflammation, could be potentially helpful to individuals with IFG and IFG/IGT to improve fasting glucose homeostasis [39,52,57]. On the other hand, plant extracts possessing mechanisms that facilitate postprandial glycemic control include inhibiting hepatic gluconeogenesis, suppressing glucagon release, enhancing the incretin effect, delaying carbohydrate digestion via inhibiting α-amylase and α-glucosidase, and slowing glucose absorption and uptake via inhibiting sodium-dependent glucose co-transporter-1 (SGLT1) and sodium-independent glucose transporter-2 (GLUT2) could potentially be utilized for individuals with IGT or IFG/IGT [40,45,57,58,59].

The question is whether plant extracts could emulate how these pharmacological agents are being categorized for a more effective, targeted clinical outcome for individuals in each prediabetes subgroup. A deeper understanding of the impact of plant extract interventions on prediabetes subgroups could enable the development of more targeted treatment strategies, with greater potential for slowing or stopping the development of T2DM.

In order to obtain results that elucidate the impact of interventions on individuals with varying degrees of dysglycemia [10,60,61], stratification based on the different glycemic profiles such as the prediabetes subgroups within the cohort is important. This will enable more specific identification of interventions appropriate for those having worsening glycemic profiles [62,63,64,65].

The present review therefore aims to (1) investigate human clinical trials that have been conducted to examine the impact of plant extracts on glycemic responses in individuals with prediabetes, and to (2) examine the effectiveness of each plant extract intervention in the prediabetes subgroups.

## 2. Human Clinical Trials Examining Effect of Plant Extracts on Glycemic Responses in the Prediabetes Cohort

Acute and chronic human clinical trials on plant extracts and involving participants with prediabetes were considered based on the ADA definition for prediabetes: IFG (FBG of 100–125 mg/dL and/or 2hPG < 140 mg/dL), IGT (2hPG of 140–199 mg/dL and/or FBG < 100 mg/dL) and IFG/IGT (FBG of 100–125 mg/dL and 2hPG of 140–199 mg/dL) [24].

Studies that included at least two glycemic measurement outcomes, such as fasting glycemic indices FBG, fasting insulin (FI), fasting C-peptide (FCP), and homeostasis model assessment-insulin resistance (HOMA-IR), and postprandial glycemic indices PG, PI, postprandial C-peptide (PCP), and glycated hemoglobin A1c (HbA1c), were considered. Only those published in English were included.

Studies that have incorporated other administered therapies, such as lifestyle modifications (e.g., physical activity) or concomitant glucose-lowering medications, or that involved fruit-based extracts, spices and traditional Chinese medicine, were beyond the scope of this review and therefore excluded.

Eleven RCT studies including one randomized, uncontrolled, parallel study covering eight different plant extracts and their impact on glycemic responses in prediabetes were identified for this review (Table 1). Two of the identified studies were acute studies and the rest, chronic studies of intervention duration ranging from four weeks to 12 weeks.

Plant extracts examined were *Artemisia princeps* Pampanini (Sajabalssuk) [66,67], *Elaeis guineensis* leaf [68], *Ficus deltoidea* leaf [68], soy (*Glycine max* (L.) Merrill) leaf [69,70], white mulberry (*Morus alba* Linn.) leaf [71,72,73,74], persimmon (*Diospyros kaki*) leaf [75], *Citrus junos* Tanaka peel [76], and *Acacia. Mearnsii* bark [77].

Ten trials involved participants with prediabetes having IFG. One trial recruited participants with prediabetes having IGT. Three trials recruited participants with prediabetes having combined IFG/IGT. All plant extracts examined were able to elicit certain significant improvement in either fasting glycemic measures such as FBG, FI, FCP and HOMA-IR, or postprandial glycemic responses such as PG, PI, PCP, as well as HbA1c in participants with prediabetes, except *Ficus deltoidea* leaf. 

**Table 1:** Human clinical trials involving plant extracts and their hypoglycemic impact in participants with prediabetes.

Plant Extract	Study Design	Total Participant Analyzed and Gender N (Male:Female)	Treatment Dose	Duration	Glycemic Measurements	Findings	Adverse Events	Reference
Sajabalssuk (*Artemisia princeps* Pampanini)	RCT, parallel study	Prediabetes IFG 99 (42:57)	Placebo, positive control or 3000 mg/day	9 weeks	FBG, FI, HOMA-IR, HbA1c, lipid profile (TG, TC, HDL, non-HDL, HTR, AI and PL), SBP, DBP, BMI, WHR, BFP, ALT and AST	Significant reduction in FBG and HbA1c compared to positive control, placebo and baseline. Significant reduction in HOMA-IR compared to placebo but not to positive control or baseline. No significant change in FI compared to positive control, placebo and baseline. Significant increase in HDL and decrease in non-HDL compared to positive control, placebo and baseline. Significant reduction in TC compared to positive control and baseline but not placebo. No significant changes in TG, HTR, AI, PL, SBP and DBP compared to positive control, placebo and baseline. No significant changes in BMI, WHR, BFP, ALT and AST compared to positive control, placebo and baseline.	Nil	[66]
Sajabalssuk (*Artemisia princeps* Pampanini)	RCT, parallel study	Prediabetes IFG and borderline diabetic 80 (51:29)	Placebo or positive control	8 weeks	FBG, FI, FCP, HOMA-IR, glucagon, HbA1c, FFA, ALT, AST, SBP and DBP	Significant reduction in FBG and HbA1c with both doses compared to baseline. No significant changes in FI, FCP, HOMA-IR, glucagon and DBP with both doses compared to baseline. Significant reduction in FFA and SBP with higher dose (4000 mg/day) compared to baseline. Significant reduction in AST with both doses compared to baseline and a significant reduction in AST with lower dose (2000 mg/day) compared to positive control, but no significant change in ALT with both doses compared to baseline.	Nil	[67]
			2000 mg/day					
			4000 mg/day					
*Elaeis guineensis* leaf	Randomized, parallel study	Prediabetes IFG 28 (gender not specified)	500 mg/day	8 weeks	FBG, FI, insulin sensitivity (%), HOMA-IR, PG AUC, PI AUC, BW and WC	Significant reduction in FBG, FI, insulin sensitivity (%) and WC compared to baseline, but no significant changes in HOMA-IR, PG AUC, PI AUC and BW compared to baseline.	Light-headedness (*n* = 1)	[68]
			1000 mg/day			Significant reduction in PG AUC, PI AUC and WC compared to baseline but no significant changes to FBG, FI, HOMA-IR, insulin sensitivity (%) and BW compared to baseline.		
*Ficus deltoidea* leaf	Randomized, parallel study		1000 mg/day			No significant changes observed.		
Soy (*Glycine max* (L.) Merrill) leaf	RCT, parallel study	Overweight and prediabetes IFG 30 (16:14)	Placebo or 2000 mg/day	12 weeks	FBG, FI, HOMA-IR, HbA1c, BW, BMI, WC, WHR, BFP, lipid profile (TG, TC, HDL, LDL, HTR, and AI), ALT, AST, SBP and DBP	Significant reduction in FBG, HOMA-IR, HbA1c, WC, BFP, TG, AI, ALT and AST compared to placebo but not when compared to baseline. Significant increase in HDL and HTR compared to placebo but not when compared to baseline. No significant changes in FI, BW, BMI, WHR, TC, LDL, SBP and DBP compared to placebo and baseline.	Nil	[69]
Pterocarpan-high Soy (*Glycine max* (L.) Merrill) leaf	RCT, parallel study	Overweight and obese, with borderline metabolic syndrome and prediabetes IFG 49 (11:38)	Placebo or 2000 mg/day	12 weeks	FBG, FI, HOMA-IR, HbA1c, BW, BMI, BFP, WHR, lipid profile (TG, FFA, TC, HDL, non-HDL, LDL, and AI), SBP, DBP, PAI-1, TNF-α, IL-6, MCP-1, adiponectin, and leptin, AST and ALT	Significant reduction in HOMA-IR and HbA1c compared to placebo and baseline. Significant reduction in FBG, FI, TC and SBP compared to baseline but not when compared to placebo. No significant changes to BW, BMI, BFP, WHR, DBP, AST and ALT compared to placebo and baseline. No significant changes to lipid profile except significant reductions in FFA and non-HDL compared to placebo and baseline. No significant changes to plasma adipokine and cytokine levels except significant reductions in PAI-1 and TNF-α compared to placebo and baseline, and significant reduction in IL-6 compared to baseline.	Nil	[70]
White mulberry (*Morus alba* Linn.) leaf and white kidney bean extract	RCT, parallel study	Prediabetes, IFG 65 (25:40)	1500 mg (500 mg mulberry extract with 10% DNJ, 1000 mg white kidney bean extract)	Acute	PG iAUC, PI iAUC, PCP iAUC	Significant reduction in PG iAUC, PI iAUC and PCP iAUC compared to control group in the acute study.	Not reported	[74]
			4500 mg/day	4 weeks		No significant changes to PG iAUC, PI iAUC, PCP iAUC HOMA-IR, HbA1c, and GSP compared to control group in the chronic study		
White mulberry (*Morus alba* Linn.) leaf and onion extract	RCT, parallel study	Prediabetes IFG 46 (5:41)	Placebo or cooked rice coated with extract (8.8 mg DNJ)	Acute	PG and PG AUC	Significant reduction in PG and PG AUC compared to placebo.	Not reported	[71]
White mulberry (*Morus alba* Linn.) leaf	RCT, parallel study	Prediabetes IFG 65 (43:22)	Placebo or extract with 6 mg DNJ	12 weeks	FBG, FI, GA, 1,5AG, HbA1c	Significant reduction in HbA1c from week 4 and GA from week 8 compared to baseline, but not when compared to placebo. No significant changes in FBG and FI compared to baseline and placebo. Significant increase in 1,5 AG from week 4, 8 and 12 compared to baseline, and overall significant increase compared to placebo.	Nil	[72]
White mulberry (*Morus alba* Linn.) leaf	RCT, parallel study	Prediabetes IFG 38 (15:23)	Placebo or 5000 mg/day (18 mg DNJ)	4 weeks	PG and PG iAUC, PI and PI iAUC, PCP and PCP iAUC, ALT and AST	Significant reduction in PG and PI only at 30 min compared to placebo. Significant reduction in PCP at 30 and 60 min compared to placebo. No significant changes in PG iAUC, PCP iAUC, ALT and AST but only PI iAUC was significantly lower than placebo.	Nil	[73]
Persimmon (*Diospyros kaki*) leaf	RCT, parallel study	Prediabetes IGT 68 (gender not specified)	Placebo or 2000 mg/day	8 weeks	PG	Significant reduction in PG compared to placebo.	Not reported	[75]
*Citrus junos* Tanaka peel	RCT, crossover study	Prediabetes IFG/IGT 35 (gender not specified)	Placebo, or 4250 mg/day	8 weeks	FBG, FI, FCP, PG, HOMA-IR	Significant reduction in FBG, FI and HOMA-IR compared to placebo but not when compared to baseline. No significant change in PG compared to placebo or baseline. No significant change in FCP when compared to placebo but significant reduction in FCP when compared to baseline.	Nil	[76]
*Acacia. Mearnsii* bark	RCT, parallel study	Prediabetes, IFG/IGT 34 (26:8)	Placebo, or 250 mg/day	8 weeks	FBG, FI, HOMA-IR, PG and PG AUC and PI and PI AUC and HbA1c	Significant reduction in PG at 90 min and PI at 90 and 120 min compared to baseline. Significant reduction in PG at 120 min and PI at 90 min after 8 weeks compared to placebo. No significant changes in PG AUC and PI AUC compared to placebo but a significant reduction compared to baseline after 8 weeks. No significant changes in FBG, FI, HOMA-IR and HbA1c after 8 weeks compared to placebo and baseline.	Nil	[77]
White mulberry (*Morus alba* Linn.) leaf	RCT, crossover study	Prediabetes IFG/IGT 10 (8:2)	Placebo	Acute	PG and PI	Not applicable.	Nil	[72]
			Extract with 3 mg DNJ			No significant change in PG compared to placebo but a significant reduction in PI at 30 min compared to placebo.		
			Extract with 6 mg DNJ			Significant reduction in PG at 30 min and significant reduction in PI at 30 min compared to placebo.		
			Extract with 9 mg DNJ			Significant reduction in PG at 30 min and significant reduction in PI at 30 min compared to placebo.		

ALT: alanine aminotransferase; AI: atherogenic index; AST: aspartate aminotransferase; BFP: body fat percentage; BMI: body mass index; BW: body weight; DBP: diastolic blood pressure; DNJ: 1-deoxynojirimycin; FBG: fasting blood glucose; FCP: fasting C-peptide; FFA: free fatty acid; FI: fasting insulin; GA: glycated albumin; GSP: glycated serum protein; HbA1c: glycated hemoglobin A1c; HDL: high-density lipoprotein cholesterol; HOMA-IR: homeostasis model assessment-insulin resistance; HTR: high-density lipoprotein cholesterol (HDL) to total cholesterol (TC) ratio; IFG: impaired fasting glucose; IGT: impaired glucose tolerance; IFG/IGT: combined impaired fasting glucose and impaired glucose tolerance; IL-6: interleukin-6; LDL: low-density lipoprotein cholesterol; MCP-1: monocyte chemotactic protein-1; PAI-1: plasminogen activator inhibitor-1; PCP: postprandial C-peptide; PCP iAUC: incremental area under the curve of postprandial C-peptide; PG: postprandial glucose; PG AUC: area under the curve of postprandial glucose; PG iAUC: incremental area under the curve of postprandial glucose; PI: postprandial insulin; PI AUC: area under the curve of postprandial insulin; PI iAUC: incremental area under the curve of postprandial insulin; PL: phospholipid; SBP: systolic blood pressure; TC: total cholesterol; TG: triglyceride; TNF-α: tumor necrosis factor-α; WC: waist circumference; WHR: waist-hip ratio; 1,5AG: 1,5-anhydroglucitol.

## 3. Effectiveness of Plant Extracts on Glycemic Responses in the Prediabetes Subgroups

Table 2 summarizes the significant changes in glycemic clinical outcomes of the interventions with the plant extracts based on each subgroup.

### 3.1. Hypoglycemic Effects of Plant Extracts on Impaired Fasting Glucose (IFG)

#### 3.1.1. *Artemisia princeps* Pampanini

*Artemisia princeps* Pampanini (*A. princeps*) belongs to one of the 500 plants under the genus Artemisia, and is commonly found in China, Korea and Japan [78,79], and has been used to treat diabetes [80,81]. High concentrations of antioxidants and flavonoids such as eupatilin and jaceosidin have likely contributed to its anti-diabetic effects [78,79,82,83]. *A. princeps* has been shown to be well tolerated with no reported side effects nor any adverse effects on the liver at a concentration of 3000 mg/day for nine weeks [66], and up to 4000 mg/day for eight weeks in humans with IFG [67].

The Korean *A. princeps* or Sajabalssuk extract (3000 mg/day) has also been examined in a prediabetes cohort for its glucose-lowering effects [66]. There were significant reductions in FBG compared to placebo and positive control after nine weeks of intervention, thus restoring normal FBG levels. Sajabalssuk extract also significantly decreased HbA1c and insulin resistance (HOMA-IR) with improvement in high-density lipoprotein (HDL) cholesterol level (*p* < 0.05) compared to control [66].

An earlier study conducted by the same group also showed significant reductions in FBG and HbA1c at both doses (2000 and 4000 mg/day) in participants with IFG and borderline T2DM (FBG 123.3 ± 5.7–125.8 ± 6.1 mg/dL) compared to participant baseline after eight weeks of intervention [67]. High dose (4000 mg/day) of the extract was also able to significantly decrease plasma free fatty acid (FFA) levels (*p* < 0.05) compared to participant baseline.

These chronic studies demonstrated that *A. princeps* (Sajabalssuk) extract was able to improve fasting glycemic responses in IFG participants. The clinical outcomes were supported by mechanistic studies done on animal models that elucidated reduced hepatic gluconeogenesis by *A. princeps* by up-regulating hepatic glucokinase (GK) and down-regulating glucose 6-phosphatase (G6Pase) activity [78], as well as phosphoenol-pyruvate carboxykinase (PEPCK), whist not having an effect on α-glucosidase inhibition [84].

#### 3.1.2. *Elaeis guineensis* Leaf

*Elaeis guineensis* (*E. guineensis*) leaf comes from oil palm and is commonly found in Malaysia, Thailand, Indonesia, Africa and South America [68,85,86]. It has been known to contain high levels of antioxidant activity rich in phenolic compounds such as catechin, apigenin and luteolin [87,88], and in vitro and animal studies have elucidated *E. guineensis* to be beneficial for metabolic syndrome and T2DM by promoting vascular relaxation and reducing inflammation and lipid oxidation [85,87,89,90,91].

Kalman and group investigated the hypoglycemic effects of *E. guineensis* leaf extract at two doses (500 and 1000 mg) in participants with IFG for eight weeks [68]. *E. guineensis* leaf extract at lower dose (500 mg) was able to significantly improve FBG (*p* = 0.02), fasting insulin (FI) (*p* = 0.04), and HOMA-IR (*p* = 0.03) compared to participant baseline levels. In contrast, the higher dose (1000 mg) was only able to significantly improve PG and PI responses (*p* = 0.046 and *p* = 0.006, respectively) compared to participant baseline levels. Having no placebo group was a limitation of the study. The improvement in glycemia in participants may be attributed to the reduction in oxidative stress [85].

Due to the paucity of clinical data regarding *E. guineensis* leaf, more research is required to investigate the glucose-lowering potential of *E. guineensis* leaf in people with prediabetes.

#### 3.1.3. *Ficus deltoidea* Leaf

*Ficus deltoidea* (*F. deltoidea*) belongs to the Moraceae plant family and is native to the Malayan Archipelago [92]. It is high in phenolic content such as flavan-3-ol monomers, catechin and afzelechin and antioxidant activity [93,94]. In the past decade in vitro and animal studies have shown *F. deltoidea* as a potential anti-diabetic treatment owing to its glucose-lowering effects and its ability to stimulate insulinotropic activity and glucose uptake [92,95,96,97,98,99,100]. *F. deltoidea* leaves have been toxicologically examined [96,97,100,101] and generally considered to be safe for human consumption [102].

There was only one 8-week prospective, randomized, double-blind, parallel study conducted investigating the impact of a single dose of *F. deltoidea* leaf extract (1000 mg) on individuals with IFG [68]. No significant changes in fasting and postprandial glucose and insulin responses were observed. Nonetheless, a number of in vitro and animal studies have elucidated that *F. deltoidea* leaf extract was able to suppress gluconeogenesis and stimulate insulin secretion to improve fasting glycemia, [95,103,104,105] and exhibited inhibitory action on α-amylase and α-glucosidase activities [99,100,106]. This therefore warrants further study investigating the potential impact of different doses on glycemic in humans.

#### 3.1.4. Soy (*Glycine max* (L.) Merrill) Leaf

Soy (*Glycine max* (L.) Merrill) leaf is common in Korea and Japan [107,108,109]. Soy leaf is rich in polyphenols such as kaempferol glycosides, coumestrol and pterocarpan [108,109,110,111], which have been shown to contain anti-diabetic properties [108,111,112]. Soy leaves at a concentration of 2000 mg/day for 10 weeks in overweight individuals has been shown to have no effects on the liver or other adverse side effects [113].

Choi and colleagues (2014) showed that consuming soy leaf extract (2000 mg/day) for 12 weeks led to significant reductions in baseline-adjusted FBG, HOMA-IR, HbA1c and lipid profile in overweight participants with IFG compared to placebo (*p* < 0.05) [69]. This finding was in agreement with another RCT investigating the impact of pterocarpan-high soy leaf extract (2000 mg/day) for 12 weeks on glucose tolerance in overweight and obese IFG participants with borderline metabolic syndrome [70]. Significant reductions in HbA1c, HOMA-IR, FFA and non-HDL cholesterol were observed in the intervention compared to control group. FBG and FI were also reduced after intervention compared to participant baseline.

The clinical outcomes suggest that that the intervention with soy leaf extract could potentially benefit those with IFG as seen in the improvements in fasting glycemic indices (FBG and HOMA-IR), with the addition of improved long-term glycemic measurement, HbA1c and improved lipid profile. Animal studies have elucidated that the underlying mechanisms observed in the glycemic improvements might be due to the regulation of β-cell proliferation, suppression of hepatic gluconeogenesis and lipid accumulation, and stimulation of insulin [107,109,114,115].

#### 3.1.5. *White mulberry* Leaf

White mulberry (*Morus alba* Linn.) leaf comes from the mulberry tree belonging to the family Moraceae, native to Korea, Japan and China but also widely cultivated in other parts in Europe [116]. Mulberry leaf has been extensively studied and reviews have been written regarding its hypoglycemic effects, in particular, its inhibition on α-glucosidase [116,117,118].

A variety of polyphenols, such as quercetin, chlorogenic acid and rutin, and nitrogen-containing glucose analog 1-deoxynojirimycin (DNJ) contained in mulberry leaf, contribute to the hypoglycemic effects observed [119,120,121,122,123,124]. DNJ has been shown as a strong α-glucosidase inhibitor due to its size and structural similarity to glucose [125,126] and has been used to standardize mulberry leaf extracts, with other phenolic components in the leaf contributing to its combined inhibitory action [71,119,125,127,128,129,130].

Mulberry leaf extract has also been tested toxicologically in humans. A concentration of 3600 mg/day of mulberry leaf extract for 38 days also did not show any adverse effects in healthy participants [131]. Another study supported the mulberry leaf extract (3000 mg/day for three months) to be safe for consumption for diabetic participants [132].

Considerable human studies have further elucidated the hypoglycemic effects of mulberry leaf extract in healthy participants, with fewer studies on prediabetes and T2DM [71,72,73,129,131,132,133,134,135,136,137,138,139]. Liu and colleagues (2020) investigated the acute hypoglycemic effects of an extract mixture of mulberry leaf and white kidney bean in participants with IFG [74]. A significant reduction in glycemic responses of incremental area under the curve (iAUC) such as PG iAUC, PI iAUC and PCP iAUC was observed compared to control. However, the glycemic improvements could not be solely attributed to mulberry leaf extract as the intervention mixture also contained white kidney bean extract. In contrast, the same study did not observe similar improvements in a 4-week chronic trial [74]. 

Hwang and co-workers (2016) investigated the impact of 50% ethanolic extract of mulberry leaf (20% in mixture) with onion extract coated on 75 g cooked rice (11.77 ± 1.67 mg DNJ/100 g rice) and observed an improvement in PG (*p* < 0.05) and postprandial glucose area under the curve (PG AUC) (*p* < 0.001) after an oral glucose tolerance test (OGTT) (75 g cooked rice) in participants with IFG compared to the placebo group [71]. The addition of a water extract of onion in the intervention mixture might have contributed to the glycemic improvements but no further information was provided in the study regarding its impact on glycemia [71].

Asai and co-workers (2011) observed a significant increase in serum 1,5-anhydroglucitol (1,5-AG) concentration, an indication of reduced postprandial hyperglycemic spikes, in participants with IFG after consuming mulberry leaf (6 mg DNJ) for 12 weeks (*p* < 0.001) in comparison to control [72]. However, no significant changes were found in FBG, FI, HbA1c and glycated albumin (GA) concentrations compared to the placebo, but HbA1c was significantly reduced from 4-week onwards within the intervention group compared to participant baseline (6.0 ± 0.4% vs. 5.9 ± 0.3%, *p* < 0.05).

Kim and colleagues (2014) investigated the impact of 4-week mulberry leaf extract (5000 mg/day, 0.36% or 18 mg DNJ) in IFG participants and demonstrated significant reductions in PG, PI and postprandial C-peptide (PCP) especially at 30 min post-load compared to placebo [73]. However, no significant changes were found in FBG, FI and HbA1c compared to the placebo.

Studies of mulberry leaf extract on healthy participants and individuals with prediabetes or T2DM have consistently shown non-significant changes in FBG and FI [72,73,131,132,138,139]. This may suggest that mulberry leaf extract, which is functionally similar to acarbose, may be more beneficial for individuals with IGT due to its inhibitory action on digestive enzyme (α-glucosidase) post-load.

Future studies of mulberry leaf could ascertain the inhibition of α-glucosidase in participants with prediabetes using hydrogen tests and starch ^13^C breath test that have been conducted in healthy and T2DM participants to indicate carbohydrate indigestion [134,135,136,139].

### 3.2. Hypoglycemic Effects of Plant Extracts on Impaired Glucose Tolerance (IGT)

#### Persimmon Leaf

Persimmon (*Diospyros kaki*) leaf belongs to the family of Ebenaceae and has been traditionally used in Japan, South Korea and China as a folk medicine [140]. The persimmon leaf has shown to possess anti-oxidative properties mediated by its rich phenolic concentration [141,142,143]. Phenolic compounds such as triterpenoids isolated from persimmon leaf have been shown to exhibit anti-diabetic properties via inhibiting protein tyrosine phosphatase 1B (PTP1B) activity (>80% inhibition at 30 μg/mL) [144]. Vomifoliol, which is found in persimmon leaf, has been identified as a potent a α-glucosidase inhibitor and an enhancer of peripheral glucose utilization [145].

Khan and colleagues (2017) demonstrated consuming 2000 mg of persimmon leaf extract for eight weeks in IGT participants led to significant PG reduction in the intervention group compared to control (*p* = 0.029) [75]. Within the same study, samples of saliva, urine and serum collected from a subgroup of five participants with combined IFG/IGT were analyzed for potential protein markers of persimmon leaf treatment. Outcomes showed Tamm–Horsfall protein, uromodulin, SPARC-like protein 1 precursor (SPARCL1) and Complement C7 were down-regulated while Ezrin was up-regulated, indicating ameliorating effects of persimmon leaf on glycemia [75].

In vitro and animal studies on persimmon leaf have elucidated the mechanistic action of α-amylase and α-glucosidase inhibition [146,147,148], which might have led to the PG reduction observed in the IGT participants due to reduced carbohydrate digestion [75]. Another mechanism of action demonstrated by persimmon leaf might be the inhibition Na+/glucose co-transporter (SGLT1) as the final stage of glucose absorption, as demonstrated by significant reductions in PG in rats after glucose loading [148].

### 3.3. Hypoglycemic Effects of Plant Extracts on Combined Impaired Fasting Glucose and Impaired Glucose Tolerance (IFG/IGT)

#### 3.3.1. *Citrus junos* Tanaka Peel

The *Citrus junos* Tanaka (*C. junos*) fruit, also known as yuja or yuzu, is a yellow citrus fruit easily obtainable in Japan, Korea and China, and contains a high concentration of phenolic content and vitamin C compared to the flesh [149,150,151,152,153]. The major phenolic compounds hesperidin and naringin [149], which have been known for improving glycemia [154].

Hwang and co-workers (2015) determined the impact of *C. junos* peel extract on glycemic responses in participants with combined IFG/IGT [76]. After eight weeks of intervention (4250 mg/day), the intervention group showed significantly reduced FBG (*p* = 0.049), FI (*p* = 0.038) and HOMA-IR (*p* = 0.019) compared to the placebo group. C-peptide in the intervention group was also marginally reduced (*p* = 0.057) but no significant improvement in PG compared to placebo. The study showed that *C. junos* peel could only improve fasting glycemic indices in combined IFG/IGT participants [76]. The hypoglycemic mechanism might be attributed to increased glucose uptake via increased insulin action in the peripheral tissues [152].

#### 3.3.2. *Acacia. mearnsii* bark

*Acacia. mearnsii* (*A.mearnsii*) bark from the black wattle tree of the legume family has been gaining attention for its anti-diabetic effects [155,156]. Its anti-diabetic potential may be attributed to the abundant antioxidants and proanthocyanidins, such as catechin-like flavan-3-ols, and in particular, robinetinidol and fisetinidol present in the bark [155].

An 8-week consumption of *A. mearnsii* bark (1000 mg/day) led to significant reduction in PG at 120 min (*p* = 0.013) and PI at 90 min (*p* = 0.032) compared to placebo in participants with combined IFG/IGT [77]. There was also a significant reduction in glucose at 90 min (*p* = 0.014), and a reduction in insulin concentration at 90 and 120 min (*p* = 0.002 and *p* = 0.004, respectively), with an overall reduction in PG AUC and postprandial insulin area under the curve (PI AUC) (*p* = 0.018 and *p* = 0.009, respectively) within the intervention group. However, there was no change in fasting glycemic measures such as FBG, FI, HOMA-IR and HbA1c compared to the placebo. Mechanistic in vitro studies indicate that the postprandial hypoglycemic effects of *A. mearnsii* bark could be due to inhibition of the digestive enzymes α-amylase and α-glucosidase [155,156,157,158,159,160,161]. This study suggests that *A. mearnsii* bark preferentially improved postprandial glycemic responses instead of fasting glycemic responses in IFG/IGT participants.

#### 3.3.3. *White mulberry* Leaf

The mulberry leaf extract was also examined in participants with combined IFG/IGT by Asai and co-workers (2011) [72]. They found that the extract (3, 6 or 9 mg DNJ) significantly reduced acute PG responses in a dose-dependent manner compared to the placebo (*p* = 0.006). This indicates that mulberry leaf may preferentially improve postprandial glycemic responses in individuals with IFG/IGT. As discussed earlier in Section 3.1.5, mulberry leaf extract was shown to improve postprandial glycemic responses in IFG participants as well.

## 4. Does Each Prediabetes Subgroup Benefit from Different Plant Extracts?

Phenolic-rich plant extracts have been known to improve glucose regulation in individuals with prediabetes; however, not all plant extracts may benefit both fasting and postprandial hyperglycemia.

Table 2 shows how the different plant extracts can be more appropriately used for individuals with IFG or IGT, based on whether they could improve fasting or postprandial glycemic responses, respectively.

Plant extracts that were able to demonstrate improvements in fasting glycemic indices such as FBG, FI and HOMA-IR were categorized as being useful for IFG. In contrast, plant extracts that demonstrated to improve postprandial glycemic indices such as PG and PI were grouped as being helpful for IGT. This is likely due to the different phenolic structures of each plant extract that enables varying kinds of hypoglycemic mechanisms of action [39,44,162,163].

Table 3 summarizes how the plant extracts that have been discussed can help individuals with IFG or IGT. Individuals with IFG/IGT are likely to benefit from both categories of glycemic improvement.

## 5. Strengths and Limitations

The merits of the present review were the inclusion of a range of human clinical trials investigating plant extracts with promising hypoglycemic potential in individuals with prediabetes, and examination of the effectiveness of each plant extract intervention on the three prediabetes subgroups.

However, the review is not without limitations. The review has included single dose studies with some studies having only small sample sizes. More human studies are necessary to ascertain the hypoglycemic impact of the plant extracts in people with prediabetes.

Most of the studies included did not clearly differentiate between the subgroups of prediabetes during recruitment. For example, nine studies that were included have only measured FBG to recruit participants with IFG; however, these participants might also have IGT, but participant baseline PG during screening was not measured. This limitation highlights the importance of measuring both fasting and postprandial indices during screening visits in future studies and for the purpose of classifying participants into the different prediabetes subgroups.

Additionally, owing to the lower reproducibility with current measurements using FBG and 2hPG, caution should be exercised when classifying individuals into IFG or IGT based on a single test [11,164]. However, the cost and practicality may need to be considered.

Furthermore, studies have not included both fasting and postprandial measurements after intervention. For example, the study of persimmon leaf extract only measured postprandial glycemic response such as PG in IGT participants. On the other hand, studies of Sajabalssuk and soybean leaf extract only measured fasting glycemic responses such as FBG and FI without investigating postprandial glycemic measures in IFG participants. Therefore, the extensive impact of the plant extracts on both fasting and postprandial glycemia could not be known.

Most of the studies included in this review did not take into account possible changes in β-cell function, insulin sensitivity and changes to lipid metabolism. This is important because the preservation or restoration of β-cell function is pivotal in slowing or halting the progression of prediabetes into T2DM [165,166,167,168]. Dyslipidaemia often occurs in prediabetes and may gradually impair insulin signaling and function, and therefore is an important endpoint measurement in interventions [169].

## 6. Conclusions

This review has explored a new perspective in viewing how nutritional interventions could cater to the different prediabetes subgroups of IFG, IGT and IFG/IGT. Among these studies, interventions with plant extracts have elucidated preferential improvements of glycemic measurements in either the fasting or postprandial state, or both, which can become beneficial for IFG, IGT or IFG/IGT, respectively. Moreover, individuals with IFG/IGT are likely to benefit from plant extracts providing either fasting or postprandial glycemic improvements. Future research on prediabetes should look into obtaining both fasting and postprandial measurements at screening and during intervention to enable participant classification into the distinct subgroups and to determine the respective impact of plant extracts on fasting and postprandial glycemia. This could allow for more tailored treatments with optimal glycemic outcomes that are specific for each of the prediabetes subgroups. Furthermore, the plant extracts discussed in this review warrant further study into their pharmacokinetic and pharmacodynamic properties along with their safety profiles for higher doses and chronic use. This will ensure the plant extracts are better characterized and standardized for use as adjuncts for prediabetes treatment.

## Figures and Tables

**Table 2:** Changes in glycemic clinical outcomes of human clinical trials in participants with prediabetes classified by their subgroups. The significant outcomes are presented in sequence first as comparison with control, then comparison within intervention group.

Plant Extract	Dose	Total Phenolic Content	Bioactive Compound Concentration	Fasting State				Postprandial State			HbA1c	Reference
				FBG	FI	FCP	HOMA-IR	PG/PG AUC	PI/PI AUC	PCP/PCP AUC		
Human clinical trials on impaired fasting glucose (IFG)												
*Artemisia princeps* Pampanini (Sajabalssuk)	3000 mg/day	Total phenolic 232.4 mg GAE/g Total flavonoid 105.2 mg QE/g	741.2 mg eupatilin /100 g, 610.3 mg jaceosidin /100 g	↓, ↓	-, -	na	↓, -	na	na	na	↓, ↓	[66]
*Artemisia princeps* Pampanini (Sajabalssuk)	2000 mg/day	Total phenolic 232.4 mg GAE/g Total flavonoid 105.2 mg QE/g	741.2 mg eupatilin /100 g, 610.3 mg jaceosidin/100 g	na, ↓	na, -	na, -	na, -	na	na	na	na, ↓	[67]
	4000 mg/day			na, ↓	na, -	na, -	na, -	na	na	na	na, ↓	
*Elaeis guineensis* leaf	500 mg/day	Not reported	Not reported	na, ↓	na, ↓	na	na, -	na, -	na, -	na	na	[68]
	1000 mg/day			na, -	na, -	na	na, -	na, ↓	na, ↓	na	na	
*Ficus deltoidea* leaf	1000 mg/day	Not reported	Not reported	na, -	na, -	na	na, -	na, -	na, -	na	na	
Soy (*Glycine max* (L.) Merrill) leaf	2000 mg/day	Total phenolic 54.1 ± 0.5 mg GAE/g Total flavonoid 90.2 ± 1.3 mg QE/g	2.09 ± 0.04 mg 6”-O-malonygenistin/g, 1.48 ± 0.03 mg coumestrol/g	↓, -	-. -	na	↓, -	na	na	na	↓, -	[69]
Pterocarpan-high Soy (*Glycine max* (L.) Merrill) leaf	2000 mg/day	Total phenolic 136.7 ± 0.0 mg GAE/g Total flavonoid 77.3 ± 0.2 mg QE/g	10.85 ± 0.26 μg coumestrol/mg, 5.90 ± 0.11 μg phaseol/mg	-, ↓	-, ↓	na	↓, ↓	na	na	na	↓, ↓	[70]
White mulberry (*Morus alba* Linn.) leaf and white kidney bean extract	1500 mg	Total phenolic 46.7 mg GAE/g Total flavonoid 2.7 mg QE/g	25 mg DNJ/1.5 g (10% DNJ)	na	na	na	na	↓	↓	↓	na	[74]
	4500 mg/day			na, na	na, na	na, na	-, na	-, na	-, na	-, na	-, na	
White mulberry (*Morus alba* Linn.) leaf and onion extract	Cooked rice coated with extract (8.8 mg DNJ)	Not reported	11.77 ± 1.67 mg DNJ/ 100 g cooked rice	na	na	na	na	↓	na	na	na	[71]
White mulberry (*Morus alba* Linn.) leaf	Extract with 6 mg DNJ	Not reported	6 mg DNJ	-, -	-, -	na	na	na	na	na	-, ↓	[72]
White mulberry (*Morus alba* Linn.) leaf	5000 mg/day (18 mg DNJ)	Not reported	3.6 mg DNJ/g	-, na	-, na	-, na	na	↓, na	↓, na	↓, na	-, na	[73]
**Human clinical trials on impaired glucose tolerance (IGT)**												
Persimmon (*Diospyros kaki*) leaf	2000 mg/day	Not reported	7.5 mg quercetin 3-O-2”galloylglucoside and kaempferol 3-O-2” galloylglucoside/g	na	na	na	na	↓, na	na	na	na	[75]
**Human clinical trials on combined impaired fasting glucose and impaired glucose tolerance (IFG/IGT)**												
*Citrus junos* Tanaka peel	4250 mg/day	Not reported	2.7 mg rutin/100 g, 1.7 mg quercetin/100 g, 0.7 mg tangeretin/100 g, 11.6 mg naringin/100 g, 36.3 mg hesperidin/100 g	↓, -	↓, -	-, ↓	↓, -	-, -	na	na	na	[76]
*Acacia. Mearnsii* bark	250 mg/day	Not reported	250 mg acacia polyphenol	-, -	-, -	na	-, -	↓, ↓	↓, ↓	na	-, -	[77]
White mulberry (*Morus alba* Linn.) leaf	Extract with 3 mg DNJ	Not reported	3 mg DNJ	-	-	na	na	-	↓	na	na	[72]
	Extract with 6 mg DNJ		6 mg DNJ	-	-	na	na	↓	↓	na	na	
	Extract with 9 mg DNJ		9 mg DNJ	-	-	na	na	↓	↓	na	na	

DNJ: 1-deoxynojirimycin; FBG: fasting blood glucose; FCP: fasting C-peptide; FI: fasting insulin; GAE: gallic acid equivalent; HbA1c: glycated hemoglobin A1c; HOMA-IR: homeostasis model assessment-insulin resistance; IFG: impaired fasting glucose; IFG/IGT: combined impaired fasting glucose and impaired glucose tolerance; IGT: impaired glucose tolerance; PCP: postprandial C-peptide; PCP AUC: area under the curve of postprandial C-peptide; PG: postprandial glucose; PG AUC: area under the curve of postprandial glucose; PI: postprandial insulin; PI AUC: area under the curve of postprandial insulin; RCT: randomized controlled trial; QE: quercetin equivalent; ↓: a significant decrease in the measured value (*p* < 0.05); -: no significant changes to measured value (*p* > 0.05); na: not applicable.

**Table 3:** Hypoglycemic effects of plant extracts on fasting and postprandial glycemic measurements.

Plant Extracts with Potential Hypoglycemic Effects on Fasting Glycemic Measurements
*Artemisia princeps* Pampanini (Sajabalssuk)
*Elaeis guineensis* leaf (500 mg/day)
Soy (*Glycine max* (L.) Merrill) leaf
*Citrus junos* Tanaka peel
**Plant extracts with potential hypoglycemic effects on postprandial glycemic measurements**
White mulberry (*Morus alba* Linn.) leaf
*Elaeis guineensis* leaf (Higher dose, 1000 mg/day)
Persimmon (*Diospyros kaki*) leaf
*Acacia. Mearnsii* bark

## Data Availability

Not applicable.
